# Opioid exposure during pregnancy and the risk of congenital malformation: a meta-analysis of cohort studies

**DOI:** 10.1186/s12884-022-04733-9

**Published:** 2022-05-11

**Authors:** Xinrui Wang, Yushu Wang, Borui Tang, Xin Feng

**Affiliations:** 1 Department of Pharmacy, Beijing Obstetrics and Gynecology Hospital, Capital Medical University, Beijing Maternal and Child Health Care Hospital, No. 17, Qi He Lou Street, Dongcheng District, Beijing, China; 2 Department of Pharmacy, Beijing Chao-Yang Hospital, Capital Medical University, Beijing, China

**Keywords:** Opioid, Pregnancy, Congenital malformation, meta-analysis

## Abstract

**Background:**

Opioid exposure during pregnancy has increased alarmingly in recent decades. However, the association between prenatal opioid exposure and congenital malformation risk has still been controversial. We aim to assess the association between opioid exposure during pregnancy and the risk of congenital malformations.

**Method:**

PubMed, Embase, and Cochrane library of clinical trials were systematically searched to September 13th, 2021. Cohort studies reporting risk of congenital malformation after opioid exposure compared with non-exposure during pregnancy were included. Risk of studies was appraised with the ROBINS-I tool. Meta-analysis was conducted using the random-effects model. Subgroup analyses were conducted for the primary outcome based on indication, exposed period, whether adjusted data was used, and risk of bias assessment. Meta-regression was performed to evaluate the relation of publication year.

**Main results:**

Eighteen cohort studies with 7,077,709 patients were included. The results showed a significant increase in the risk of overall congenital malformation (RR = 1.30, 95%CI: 1.11–1.53), major malformation (RR = 1.57, 95%CI:1.11–2.22), central nervous system malformation (RR = 1.36, 95% CI:1.19–1.55), and limb malformation (RR = 2.27, 95%CI:1.29–4.02) with opioid exposure during pregnancy. However, the predictive interval conveyed a different result on overall congenital malformation (95%PI: 0.82–2.09) and major malformation (95%PI: 0.82–2.09). No association between opioid exposure and overall congenital malformation in the first trimester (RR = 1.12, 95%CI:0.97–1.31) and prescribed for analgesic or antitussive treatment (RR = 1.03, 95%CI:0.94–1.13) were observed. In subgroups that study provided data adjusted for confounders (RR = 1.06, 95%CI:0.93–1.20) or identified moderate or serious risk of bias (RR = 1.00, 95%Cl: 0.85–1.16; RR = 1.21, 95%Cl: 1.60–2.68), no association was found.

**Conclusion:**

Opioid exposed in the first trimester or prescribed for analgesic or antitussive treatment did not increase the risk of overall congenital malformation. The findings should be discussed in caution considering the situation of individual patients and weigh out its potential risk of congenital malformation.

**Trial registration:**

Registration number: CRD42021279445.

**Supplementary Information:**

The online version contains supplementary material available at 10.1186/s12884-022-04733-9.

## Introduction

Opioid is frequently prescribed for pain, such as lower back pain and pelvic joint pain, to reduce perception of pain during pregnancy. Also, opioid medications, such as methadone and buprenorphine, are used to treat opioid use disorder [1]. Opioid exposure during pregnancy has increased alarmingly in recent decades [2–8]. It was reported that 21.6% of women receive an opioid prescription during pregnancy in the US, which meant up to one of five pregnant women filled an opioid prescription [4]. The prescriptions were widespread in either commercial insurance or Medicaid [5, 9]. A 2019 self-reported study found that about 7% of women reported using prescription opioid pain relievers during pregnancy [7]. On the other hand, increasing opioid use among reproductive-age women has also been widespread [9, 10]. Since unplanned pregnancies are not uncommon and many pregnancies are not recognized until a few weeks after conception [11], all women prescribed opioid at reproductive age were at potential risk [12].

The association between prenatal opioid exposure and congenital malformation risk has still been controversial. Two studies funded by the Centers for Disease Control and Prevention have set off an upsurge in studying the relationship between opioid use during pregnancy and congenital malformations [13, 14]. Some studies reported an increased risk of congenital malformations in relation to maternal opioid use [13, 15–17], while other studies have found no association [18, 19]. Specially, a systematic review from the CDC in the US reported some potential higher risk of congenital malformations related to opioid exposure during pregnancy, such as congenital malformations overall, cardiovascular malformations, oral cleft, and clubfoot [20]. However, they still reported uncertainty regarding the teratogenicity of opioids. Recently, two large population-based cohort studies have been conducted to explore the gestational opioid exposure and risk of congenital malformations in Europe and the US [21, 22]. Bateman et al. [21] reported that prescription opioids used in early pregnancy are not associated with a substantial increase in risk for most of the malformation types considered except oral clefts. Wen [22] found no excess risk for major birth defects in infants with opioid exposure in the first trimester. In contrast, a higher risk of minor congenital malformations associated with opioid use in the third trimester was found.

These findings call for the safety re-evaluation of opioid exposure during pregnancy to inform clinical practice. Therefore, we performed a meta-analysis using data from real-world cohort studies to assess the association between opioid exposure during pregnancy and the risk of congenital malformations.

## Methods

### Protocol and registration

We followed the Meta-analysis of Observational Studies in Epidemiology [23] to perform the meta-analysis. The study protocol was registered at https://www.crd.york.ac.uk/prospero/ (registration number CRD42021279445) before searching articles.

### Eligibility criteria

We used PICOS model to select the population. The inclusion criteria were: (1) cohort studies; (2) investigated opioid use during pregnancy; (3) reported both opioids-exposed and -unexposed group; (4) reported on any congenital malformations and specific congenital malformation at birth; (5) reported available data, such as odds ratio (OR), adjusted OR, risk ratio (RR), adjusted RR, hazard ratio (HR), or data to calculate RR; (6) reported outcomes including any congenital malformations, major congenital malformation, and/or sub-categories of congenital malformations. The exclusion criteria were: (1) review, systematic review and meta-analysis, conference abstract, and case report; (2) not human studies; (3) did not clarify the exposure of opioid during pregnancy; (4) overlapped data source is included.

### Search strategy

We systematically searched PubMed, Embase, and Cochrane library of clinical trials up to September 13th, 2021. The search terms were attached in Table S1.

### Selection of studies and data extraction

Two reviewers (X.W, Y.W) independently screened titles and abstracts through Endnote (version 9.3.2). Duplications were removed through Endnote and manually. We also screened the references lists of relevant reviews and articles. Any disagreement was resolved by discussion until consensus was reached or by consulting a third author (X.F).

Data were independently extracted by two investigators (X.W and Y.W) for eligible studies. Disagreements were discussed and resolved by a third author (X.F). The data obtained for each study included first author, year of publication, study setting, drug used, exposure measurement, exposed period, outcome assessment, indication, sample size, congenital malformations with their risk estimates, 95% confidence interval (CI) and 95% prediction interval (PI). The primary outcome was overall congenital malformations. The secondary outcome was organ-specific congenital malformations.

### Quality assessment

We assessed the risk of bias for each study included using the ROBINS-I tool [24], which is developed for evaluating risk of bias of interventions for non-randomized studies. The quality of each study was evaluated for the risk of bias in seven domains: (1) bias due to confounding; (2) bias in selection of participants into the study; (3) bias in classification of intervention; (4) bias due to deviations from intended interventions; (5) bias due to missing data; (6) bias in measurement of outcomes; (7) bias in selection of the reported result. The interpretations of domain level and overall judgment for risk of bias are classified as low, moderate, serious, or critical.

We evaluated the level of evidence for each outcome using Grading of Recommendations, Assessment, Development and Evaluation (GRADE) approach [25]. The results were classified as high, moderate, low, or very low.

### Statistical analysis

The meta-analysis was conducted with R (version 4.0.5). For the expected high heterogeneity in terms of the enrolled populations, DerSimonian and Laird random-effect models was used to pool RRs along with the corresponding 95% CIs. Due to the low prevalence of congenital malformation in the general population, we proposed RR, HR, and OR to be comparable. For those studies that did not report the RRs of congenital malformations, we used other risk measures, including ORs or HRs, as an approximation to the RRs. Therefore, we summarized them together using meta-analysis methods. The adjusted effect sizes were selected to pool the risk estimates preferentially. Statistical heterogeneity among studies was tested using Cochran’s Q test and the I^2^ statistic. I^2^ > 50% or *P* <  0.05 was considered to indicate significant heterogeneity. We also addressed heterogeneity by calculating the 95% prediction interval for the pooled unadjusted OR, which gives an estimate of the point at which the true effects are to be expected for 95% of similar studies that might be conducted in the future [26]. The Egger test was used to assess the funnel plot for asymmetry, indicating possible publication biases.

To explore the sources of heterogeneity, subgroup analyses were run for primary outcome based on indication (analgesic or antitussive treatment, opioid abuse or opioid abuse treatment), exposed period (first trimester, all trimesters), risk of bias assessment (moderate, serious, critical), and whether adjusted data was used (yes, no). Due to the large span of publication year of included studies, we performed random-effects meta-regression analyses by the empirical Bayes method to estimate the between-study variance and the method by Hartung and Knapp was used to adjust statistics and evaluate the relation of covariates (year of publication) on the primary outcome. To evaluate the stability of the results, sensitivity analyses were performed with the leave-one-out method.

## Results

**Fig. 1:**
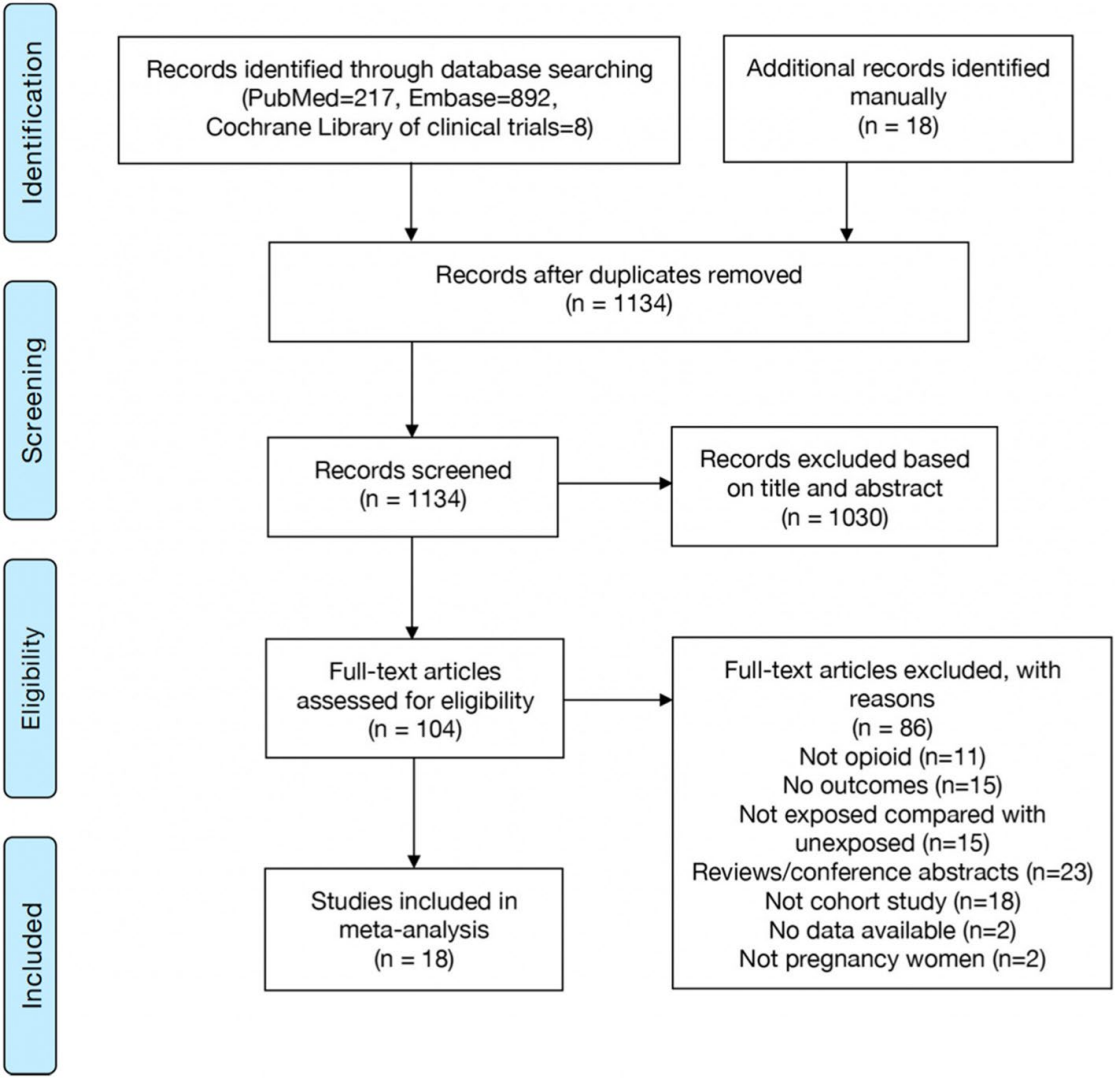
Flow-chart of studies selection

One thousand one hundred seventeen studies were identified after database searching. 18 additional records were identified manually through references lists of relevant articles. After removing the duplications, 1030 studies were excluded by screening titles and abstracts. Only 18 studies [15, 16, 19, 21, 22, 27–39] were eligible for meta-analysis after full-text assessment (Fig. 1). Characteristics of the included studies are presented in Table 1. The included studies were published between 1976 and 2021. According to the results of risk of bias assessment using ROBIN-I tool, the risk of bias of each included study ranged from moderate to critical. The results of all domains of quality assessment are summarized in Table S2. In addition, according to the GRADE approach, the overall level of evidence among all outcomes ranged from very low to moderate (Table 2).

**Table 1:** Baseline characteristic of the included studies

Author/ Year	Setting	Drug	Exposure measurement	Exposed period	Outcome assessment	Indication	Sample size (Exposed/ Not exposed)	Adjusted factors
Bateman /2021 [21]	Medicaid Analytic eXtract, 2000–2014, MarketScan Research Database, 2003–2015	Opioid prescriptions including hydrocodone, oxycodone, codeine	Drug prescription recorded in databases	First trimester	Medical records, ICD-9 code	Abdominal pain; Back/neck pain; Fibromyalgia; Dental problems; Migraine/headache; Orthopedic injury; Surgery	82,901/ 2,697,355	Indications for opioids, maternal demographic characteristics, chronic comorbidities, concomitant medication use, and general markers of the burden of illness
Wen /2021 [22]	Rhode Island Medicaid claims, 2008–2016	NA	Drug prescription recorded in databases	First/second/third trimester (available data only for first trimester)	Rhode Island birthing hospitals records, Rhode Island Birth Defects Program, ICD-9-CM code, ICD-10-CM code	Lower back pain; Headache; Chronic pelvic pain; Fibromyalgia	891 (433 in first trimester)/ 11,533	Age, obesity, multiple births, tobacco use, alcohol use, other substance abuse, type of pain, history of diabetes, hypertension, depression, anxiety, bipolar disorder, attention deficit/hyperactivity disorder, menstrual disorder, and use of comedications during the baseline or exposure trimester
Fishman /2019 [29]	Soroka Medical Center, 1999–2009	Propoxyphene, codeine, tramadol, oxycodone, fentanyl	Drug prescription recorded in databases	During the first 13 weeks of gestation	Soroka Medical Center records, registry of the Committee for Termination of Pregnancies at Soroka Medical Center, ICD-9 code	NA (Patients use illicit drugs in the present or past were excluded)	3003/98583	Maternal age, ethnic group, self-reported tobacco use during pregnancy, pre-gestational diabetes mellitus, maternal obesity, nulliparity, and folic acid intake
Kelty /2017 [16]	Health Department of Western Australia’s Monitoring of Drugs of Dependence System, 2001–2010	Buprenorphine, naltrexone, methadone	Drug prescription computerized records	During pregnancy	West Australia Register of Developmental Anomalies, ICD-9 code	Patients receiving sustained-release naltrexone implant	391/569	Maternal age at birth, number of previous pregnancies, cigarette smoking during pregnancy, and socioeconomic status
Jumah [18] /2016	Outpatient antenatal clinic records and inpatient medical records, 1 July, 2010–31 July, 2015	Buprenorphine, naloxone, illicit opioid	Medical records review	During pregnancy	NA	Patients receiving opioid agonist treatment	159/618	None
Kallen /2015	Swedish Medical Birth Register, 1997–2013	Tramadol	Structured interview	Early pregnancy	Medical Birth Register, ICD code	NA	1751/1681095	Year of delivery, maternal age, parity, smoking, and BMI before pregnancy
Norgaard /2015	Danish Medical Birth Registry, 1997–2011	Buprenorphine, methadone, heroin	Drug prescription records in Danish Register of Medicinal Product Statistics, having a relevant cisit recorded in the Registry of Drug Abusers Undergoing Treatment	During pregnancy	Danish National Registry of Patients, ICD-10 code	Opioid maintenance treatment	557/945569	Smoking and parity
Saleh Gargari /2012 [35]	Four major hospitals affiliated to Shahid Beheshti and Tehran Universities of Medical Sciences, April 1, 2004-March 31, 2009	Opium, heroin, crack, cannabis, methamphetamine	Medical records review	During pregnancy	NA	Substance abuse	439/519	Maternal age, gravidity, and parity
Greig /2012 [30]	A London teaching hospital and “Methadone Substitution Programme (MSP)” tertiary referral centre	Methadone	Liaison Antenatal Drugs and Alcohol Service (LANDS) Clinic records review	During pregnancy	NA	Problematic substance misuse	44/88	Maternal age, gravidity, and parity
Nezvalová-Henriksen /2011 [19]	Norwegian Mother and Child Cohort Study and Medical Birth Registry of Norway, 1999–2006	Codeine	Self-administered questionnaires	During pregnancy and every trimester	Medical Birth Registry of Norway	NA	2666/65316	Sociodemographic, lifestyle, medical characteristics, concomitant drug use, and factors related to delivery
Vucinovic /2008 [37]	Split University Hospital, 1997–2007	Heroin, methadone, other substance	Medical records reviews	During pregnancy	NA	Opioid addiction	86/43529	None
Cleary /2011 [15]	Coombe Women and Infants University Hospital, 2000–2007	Methadone	Antenatal records, controlled drug registers, prescription records	At delivery	EUROCAT classification system	Opiate-dependent	618/60412	Age at delivery, socioeconomic group, nationality, marital status, nulliparity, planning of pregnancy, booking gestation, receipt of publicly funded health care, number of cigarettes per day, and units of alcohol used per week before pregnancy
Kallen /2013	Swedish Medical Register, 1996–2011	Opioids	Drug prescription recorded in the Register	First trimester/second trimester/third trimester	Medical Birth Register, Register of Birth Defects, Hospital Discharge Register ICD-10	NA	7654/1369432	Year of birth, maternal age, parity, smoking in early pregnancy, BMI, and concomitant use of other drugs
Brown /1998 [27]	Wishard Memorial Hospital, July 1993–March 1996	Methadone, cocaine	Positive urine drug screen	During pregnancy	NA	Narcotic-addicted women who used cocaine; methadone maintenance treatment	64/32	Gestional age and parity
Ellwood /1987 [28]	A drug-dependency antenatal clinic during December 1983	Methadone, cocaine	Medical records review	During pregnancy	NA	Methadone maintenance treatment	182/182	None
Wilson /1981 [38]	Houston’s public maternity hospital, August 1974 and July 1977	Heroin, methadone	Structured interview and medical records review	During pregnancy	NA	Narcotic-addicted women	68/58	Maternal age, race, socioeconomic level, marital status, and duration of gestation at the time prenatal care
Ostrea /1979 [39]	Hutzel Hospital, 1973–1976	Heroin, methadone	NA	During pregnancy	NA	Clinic patients who used methadone and heroin; nonclinic patients who were on heroin	830/400	None
Stimmel /1976 [36]	NA	Heroin, methadone	Medical records review	During pregnancy	NA	Drug-addicted who used heroin; methadone maintenance treatment	85/30	None

*US* United State, *UK* United Kingdom, *NA* not available

**Table 2:** GRADE assessment on the certainty of evidence for all the outcomes

Outcomes (no. of studies)	Certainty assessment					No. of patients	Risk Ratio (95% Confidence Interval)	Certainty
	Risk of bias	Inconsistency	Indirectness	Imprecision	Other considerations			
Overall congenital malformation (13)	Serious	Serious	Serious	Not Serious	None	7,246,838	1.30(1.11,1.53)	Very low
Major malformation (9)	Serious	Serious	Serious	Not Serious	None	143,754	1.57(1.11,2.22)	Very low
Cardiovascular malformation (6)	Serious	Not Serious	Serious	Serious	None	6,186,924	1.70(0.62,4.63)	Very low
Central nervous system malformation (6)	Serious	Not Serious	Serious	Not Serious	None	4,504,346	1.07(0.90,1.26)	Low
Gastrointestinal malformation (3)	Serious	Not Serious	Not Serious	Serious	None	163,690	1.48(0.27,7.98)	Low
Ear, face, and neck malformation (2)	Serious	Not Serious	Not Serious	Serious	None	62,104	2.27(1.29,4.02)	Low
Limb malformation (2)	Not Serious	Not Serious	Not Serious	Not Serious	None	1,621,180	1.36(1.19,1.55)	Moderate
Respiratory malformation (2)	Serious	Not Serious	Not Serious	Serious	None	62,104	2.46(0.34,17.78)	Low
Musculoskeletal malformation (3)	Serious	Not Serious	Not Serious	Serious	None	163,690	1.35(0.81,2.26)	Low
Urogenital malformation (5)	Serious	Not Serious	Serious	Serious	None	1,848,287	0.93(0.65,1.33)	Very low
Orofacial clefts (4)	Not Serious	Serious	Not Serious	Serious	None	4,503,022	1.08(0.48,2.44)	Low
Neural tube defects (4)	Serious	Not Serious	Serious	Serious	None	4,442,356	0.90(0.63,1.30)	Very low
Gastroschisis (2)	Not Serious	Serious	Not Serious	Serious	None	2,841,400	2.08(0.84,5.20)	Low
Clubfoot (2)	Not Serious	Serious	Not Serious	Serious	None	6,023,252	1.28(0.82,2.00)	Low

GRADE, grading of recommendations assessment, development, and evaluation

Opioid indications were analgesic or antitussive treatment, opioid abuse, and opioid abuse treatment. The total sample size of these studies ranged from 96 to 2,780,256. Kallen 2015 and Kallen 2013 were both from the Swedish Medical Birth Register and the time covered were overlapped. Therefore, we included Kallen 2013 (reported all opioids exposure) for statistical analysis in most outcomes. Kallen 2015 were included for analysis for urogenital malformation, where Kallen 2013 did not provide available data. Sensitivity analysis and subgroup analysis was conducted only for overall congenital malformations for the few studies included in different specific congenital malformations.

### Overall congenital malformation

**Fig. 2:**
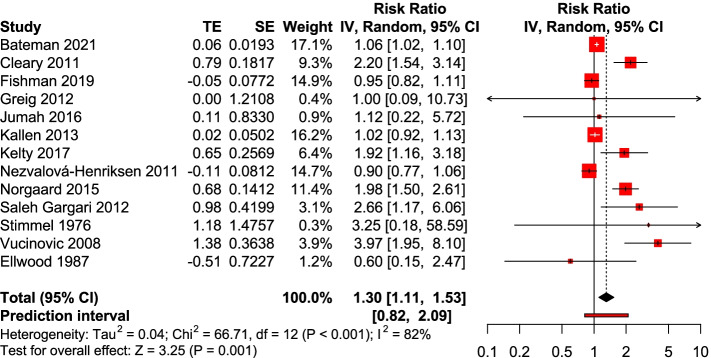
Forest plot of opioid exposure during pregnancy and the risk of congenital malformation; TE, treatment effect; SE, standard error; IV, inverse variance test; CI, confidence interval; df, degrees of freedom

Thirteen studies [15–17, 19, 21, 28–32, 35–37] reported overall congenital malformation. The results showed a significant increase in the risk of congenital malformations with opioid exposure during pregnancy (RR = 1.30, 95%CI: 1.11–1.53); however, the 95% predictive interval (95%PI: 0.82–2.09) did not show the same effect. This indicates the uncertainty of the estimates and in the conclusions, given the observed between-study heterogeneity (P<0.001, I^2^ = 82%) (Fig.2). No evident asymmetry in the funnel plot (Fig. S1).

### Organ-specific congenital malformations

**Fig. 3:**
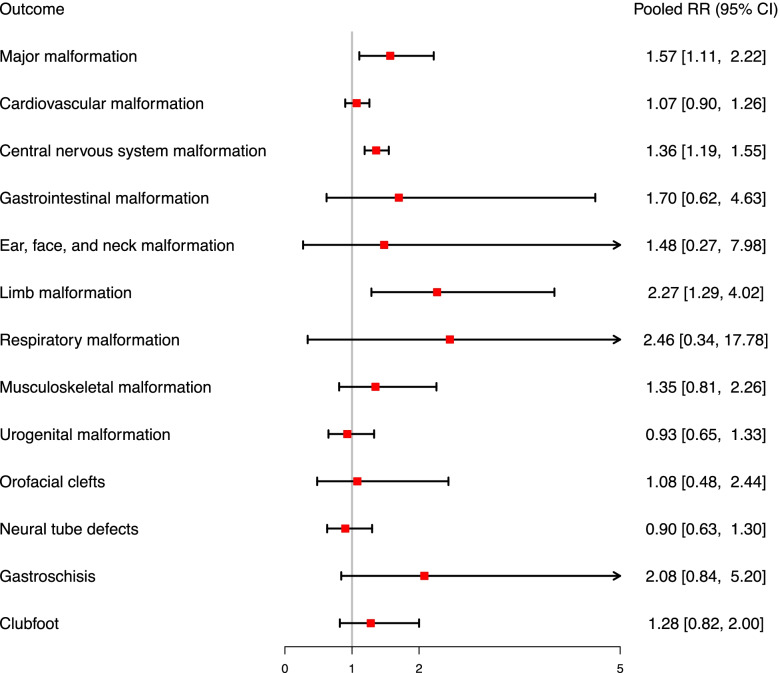
Forest plot of opioid exposure during pregnancy and the risk of specific congenital malformation; RR, risk ratio; CI, confidence interval

The summary of meta-analysis of 13 estimates, which analyzed organ-specific congenital malformations, was shown in Fig. 3. The interpretation of major malformation (RR = 1.57, 95%CI:1.11–2.22), central nervous system (CNS) malformation (RR = 1.36, 95%CI:1.19–1.55) and limb malformation (RR = 2.27, 95%CI:1.29–4.02) using the confidence interval shows a statistically significant treatment effect, whereas the predictive interval conveyed a different result on major malformation (95%PI: 0.82–2.09). No significant relationship between opioid use and cardiovascular malformation, gastrointestinal malformation, ear, face, and neck malformation, respiratory malformation, musculoskeletal malformation, urogenital malformation, orofacial clefts, neural tube defects, gastroschisis, and clubfoot were found (Fig. S2–14).

### Sensitivity analysis and meta-regression

**Fig. 4:**
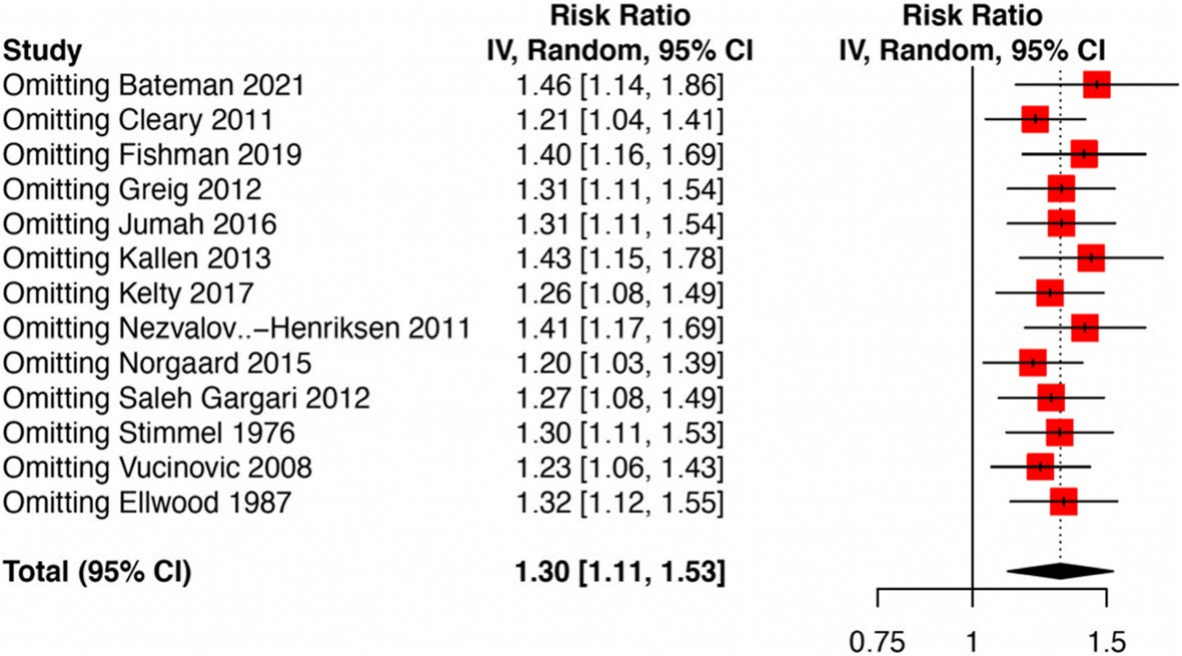
Summary of sensitivity analysis by leave-one-out method

The sensitivity analyses revealed no substantial change in the pooled risk estimates upon excluding of any single study (Fig. 4). Meta-regression analysis based on the year of publication showed no significant relationship (Fig. S15).

### Subgroup analysis

We performed subgroup analyses regarding exposed period, indication, adjusted for confounders, and risk of bias assessment by ROBINS-I (Fig. S16–20). The results were summarized in Table 3. There was no significant increased risk of overall congenital malformation among studies that specifically examined exposure to opioids in the first trimester (RR = 1.12, 95%Cl:0.97–1.31). In contrast, studies reported opioid exposure during pregnancy, no significant result was observed. When stratified by indication, studies that use opioid abuse or opioid abuse treatment as a reason for opioid exposure, the risk (RR = 2.09, 95%Cl:1.74–2.52) was significantly increased. No difference was found for those opioid use for analgesic or antitussive treatment (RR = 1.03, 95%Cl:0.94–1.13). In addition, a significant association was found in studies that used unadjusted data (RR = 2.07, 95%CI:1.60–2.68), but not in studies that provided adjusted data (RR = 1.06, 95%CI:0.93–1.20). Furthermore, studies with moderate or serious risk of bias showed no significant difference between opioid exposure and overall congenital malformation (RR = 1.00, 95%Cl: 0.85–1.16; RR = 1.21, 95%Cl: 0.90–1.63).

**Table 3:** Summary risk estimates of the relationship between opioid exposure and overall congenital malformations

	No. of study	Summary RR (95%CI)	I_**2**_ (%)	P*	P**
Exposed period					0.113
First trimester	4	1.12(0.97, 1.31)	86	0.127	
During pregnancy	9	1.72(1.04, 2.84)	81	**0.035**	
Indication					< 0.001
Analgesic or antitussive treatment	2	1.03(0.94, 1.13)	47	0.549	
Opioid abuse/opioid abuse treatment	9	2.09 (1.74, 2.52)	0	**< 0.001**	
Not reported	2	0.97 (0.87, 1.10)	42	0.661	
Adjusted for confounders					< 0.001
Yes	5	1.06 (0.93, 1.20)	82	0.364	
No	8	2.07 (1.60, 2.68)	9	**< 0.001**	
Risk of bias assessment					< 0.001
Moderate	2	1.00 (0.85, 1.16)	74	0.955	
Serious	3	1.21 (0.90, 1.63)	89	0.214	
Critical	8	2.07 (1.60, 2.68)	9	**< 0.001**	

*RR* risk ratio, *CI* confidence interval, P*, *P*-value in-subgroup; P**, *P*-value between-subgroup; **bold** indicate significant difference

## Discussion

The overall result of this meta-analysis included 18 cohort studies and demonstrated opioid exposure during pregnancy with a 1.3-fold risk of congenital malformations. Additionally, opioid use was associated with increased risks of major malformation, CNS malformation, and limb malformation with an increase of 57, 36, and 127%, respectively. We found no significant relationship between opioid use and cardiovascular malformation, gastrointestinal malformation, ear, face, and neck malformation, respiratory malformation, musculoskeletal malformation, urogenital malformation, orofacial clefts, neural tube defects, gastroschisis, and clubfoot were found. No association in subgroups that opioid was exposed in the first trimester or prescribed for analgesic or antitussive treatment. However, the positive findings were only observed in subgroups that studies provided data unadjusted for confounders or identified critical risk of bias assessment. In subgroups that study provided data adjusted for confounders or identified moderate or serious risk of bias, no association of opioid exposure and overall congenital malformation was found between exposed and not exposed group.

Opioids can compound act on the endogenous opioid system, which comprises four G protein-coupled receptors and four major peptide families. They can regulate neuronal function and neurotransmission in human brain, brain stem and other tissues, to effectively prevent the sensation of pain from being transmitted to the brain [40]. Pregnant women would experience various physiological changes in the body, such as changes in renal blood flow, gastric emptying speed, plasma protein level and apparent distribution volume, making it difficult to predict the pharmacokinetic metabolism of opioids. For instance, maternal hepatic metabolism altered in pregnancy [41, 42], affecting the pharmacokinetics of several opioids metabolized through these pathways [43]. The increase of tidal volume and respiratory rate during pregnancy may also promote the absorption of drugs into the system through the alveoli, which could amplify fetal drug exposure to inhaled opioids [44]. Moreover, the plasma albumin of pregnant women will gradually decrease and reach stabilization at the end of the first trimester in pregnancy, which will make the free fraction of high plasma protein binding drugs such as oxycodone, methadone, and fentanyl higher than non-pregnant women [45, 46]. Zagon [47] found that the opioid exposure of rats during pregnancy will reduce DNA synthesis in three germ layer organ cells which leads to fetal congenital malformation. It was proposed that exogenous opioids during the critical period might destroy the normal development process and lead to fetal congenital malformations. Nevertheless, we found a significantly increased risk of congenital malformation during pregnancy while no difference in the first trimester. More research exploring the biological mechanism of opioid exposure and congenital malformation were needed.

When stratified by opioid indications, we found opioid use for analgesic or antitussive treatment did not associate with a higher risk of congenital malformation compared with no exposure. On the contrary, patients who use opioids for abuse or opioid-dependent treatment were more likely to give birth to babies with congenital malformations. The cumulative dose of drug used varied between these indications may explain one of the reasons, which might cause increased blood drug level and risk of congenital malformations. Given that those who were addicted to opioids were at greater risk of misusing prescription opioids and might use more opioids, which is way higher than the therapeutic safe boundary [48]. Only two studies reported the dose-response relationship between opioid and congenital malformations. Wen [22] observed that overall minor birth defects showed significant dose responses in trimester 3. No evidence of increasing risk with higher cumulative opioid exposure was found for any of the primary outcomes as demonstrated by Bateman [21]. The higher dosage range of these studies varied from ≥42.25 cumulative morphine milligram equivalent (MME) to > 300 MME. More research assessing the dose-response relationship between opioid use and the risk of congenital malformations should be pursued.

After stratified by whether the study controlled for potential confounders to avoid unpredictable bias introduced by other confounders, we found significant heterogeneity between subgroups. The confounding factors included baseline characteristic such as maternal age, obesity, tobacco use, parity, and so on. Alternatively, the result of confounders adjusted studies showed no association between opioid use and overall congenital malformations, which is different from the pooled result. The potential confounders adjusted in these studies were not consistent. For example, Bateman [21] and Nezvalová-Henriksen [19] adjusted for concomitant medication use while others did not. Furthermore, in both subgroup of moderate and serious risk of bias, most of which adjusted for confounders, the result showed no association between opioid exposure and overall congenital malformation. The results still provide reasonable doubt that after adjusting some potential confounders, opioid itself did not contribute to a higher risk of overall congenital malformations.

Our findings provide evidence for health professionals to weigh the benefit of opioid along with its potential risks. Also, pregnant women, women intended to get pregnant, or reproductive-aged women at risk of any unintended pregnancy could evaluate the potential risk of opioid during pregnancy. Still, the use of opioid in some situations, especially medication assisted therapy for the treatment of substance use disorder, might provide far greater benefits than risks [49]. Our results should be treated with caution by pregnancy opioids users or potential opioid users to make the safest choice. Besides, since we detected raised risk of major congenital malformation, CNS malformation, and limb malformation, exposed pregnant women could take ultrasound examinations more frequently to detect the fetus growth, especially for CNS growth and limb growth.

Our study has several strengths. To date, this is the first meta-analysis evaluating the association between opioid exposure and the risk of congenital malformations. The meta-analysis included a large sample size of 7,077,709 patients and only cohort studies to reduce recall and selection bias. We also did comprehensive subgroup analyses to evaluate the relationship between opioid use and congenital malformations.

Our findings are also subject to several limitations. Firstly, the publication year of the studies included ranged from 1976 to 2021 and might contribute to methodologic bias. However, no significance was observed on meta-regression evaluating the relation of publication year and overall congenital malformations. Secondly, high heterogeneity was detected in most of the outcomes. The reason might be that all included studies are retrospective studies, with the potential for confounding. We performed subgroup analyses to reduce the possible influence. Besides, included studies reported congenital malformations based on several kinds of opioids. Some contained only methadone, and some investigated opioid prescriptions, including hydrocodone, oxycodone, codeine. This might contribute to the heterogeneity of the results. Thirdly, the evaluation of the prediction interval revealed that the current 95%CI produces a positive biased estimate of the overall congenital malformation and major malformation, probably due to the between-study heterogeneity, or to the very low certainty of evidence for the two outcomes. Therefore, large size studies with higher level evidence are needed. Fourthly, most of the studies considered the pregnancies as opioid-exposed by referring to prescriptions during pregnancy, it might be possible that though prescriptions were dispensed while opioids were not taken. Well-designed prospective studies are needed to affirm the findings. Fifthly, few studies reported organ-specific malformations, and the categories of malformations reported were inconsistent. For example, Cleary [15] reported 10/13 of the organ-specific malformations, Kelty [16] reported 8/13, and Brown [27] only reported 2/13. Hence, we were unable to carry subgroup analyses. More studies were needed to provide data classified by specific organs to assess the association between opioid exposure and the risk of organ-specific malformations.

## Conclusion

In conclusion, we found that maternal opioid exposure in pregnancy was associated with increased risk of major malformation, CNS malformation, and limb malformation. Opioid exposed in the first trimester or prescribed for analgesic or antitussive treatment did not increase the risk of overall congenital malformation. In studies with moderate or serious risk of bias or studies adjusted for confounders, no association was found between opioid exposure and overall congenital malformation. Therefore, the results should be interpreted in caution.

## Supplementary Information

**Additional file 1 Table S1.** Search terms. **Table S2.** Summary of risk of bias assessment using ROBINS-I tool. **Fig. S1.** Eggers’s test of studies examining the association between opioids exposure and the risk of congenital malformations. **Fig. S2.** Forest plot of association between opioid exposure and major congenital malformation. **Fig. S3**. Forest plot of association between opioid exposure and central nervous system malformation. **Fig. S4.** Forest plot of association between opioid exposure and limb malformation. **Fig. S5.** Forest plot of assoxiation between opioid exposure and cardiovascular malformation. **Fig. S6.** Forest plot of association between opioid exposure and gastrointestinal malformation. **Fig. S7.** Forest plot of association between opioid exposure and ear, face, and neck malformation. **Fig. S8.** Forest plot of association between opioid exposure and respiratory malformation. **Fig. S9.** Forest plot of association between opioid exposure and musculoskeletal malformation. **Fig. S10.** Forest plot of association between opioid exposure and urogenital malformation. **Fig. S11**. Forest plot of association between opioid exposure and orofacial malformation. **Fig. S12.** Forest plot of association between opioid exposure and neural tube defects. **Fig. S13.** Forest plot of association between opioid exposure and gastroschisis. **Fig. S14.** Forest plot of association between opioid exposure and clubfoot. **Fig. S15.** Meta-regression according to the year of publication. **Fig. S16.** Forest plot of subgroup analysis of exposed period. **Fig. S17.** Forest plot of subgroup analysis of indication. **Fig. S18:** Forest plot of subgroup analysis of adjusted for confounders. **Fig. S19.** Forest plot of subgroup analysis of risk of bias assessment.

## Data Availability

All data are available within the manuscript and supplemental materials.
