# Supplementary material for: Opioid exposure during pregnancy and the risk of congenital malformation: a meta-analysis of cohort studies
Source: BMC Pregnancy Childbirth. 2022 May 11;22:401. doi: 10.1186/s12884-022-04733-9 (PMC9097072; doi:10.1186/s12884-022-04733-9)
Supplement: Supplementary file 1 — Additional file 1 Table S1. Search terms. Table S2. Summary of risk of bias assessment using ROBINS-I tool. Fig. S1. Eggers’s test of studies examining the association between opioids exposure and the risk of congenital malformations. Fig. S2. Forest plot of association between opioid exposure and major congenital malformation. Fig. S3. Forest plot of association between opioid exposure and central nervous system malformation. Fig. S4. Forest plot of association between opioid exposure and limb malformation. Fig. S5. Forest plot of assoxiation between opioid exposure and cardiovascular malformation. Fig. S6. Forest plot of association between opioid exposure and gastrointestinal malformation. Fig. S7. Forest plot of association between opioid exposure and ear, face, and neck malformation. Fig. S8. Forest plot of association between opioid exposure and respiratory malformation. Fig. S9. Forest plot of association between opioid exposure and musculoskeletal malformation. Fig. S10. Forest plot of association between opioid exposure and urogenital malformation. Fig. S11. Forest plot of association between opioid exposure and orofacial malformation. Fig. S12. Forest plot of association between opioid exposure and neural tube defects. Fig. S13. Forest plot of association between opioid exposure and gastroschisis. Fig. S14. Forest plot of association between opioid exposure and clubfoot. Fig. S15. Meta-regression according to the year of publication. Fig. S16. Forest plot of subgroup analysis of exposed period. Fig. S17. Forest plot of subgroup analysis of indication. Fig. S18: Forest plot of subgroup analysis of adjusted for confounders. Fig. S19. Forest plot of subgroup analysis of risk of bias assessment. [file 12884_2022_4733_MOESM1_ESM.docx]

**Table S1** Search terms

| PubMed |
| --- |
| (("Analgesics, Opioid"[Mesh]) OR opioid OR opiate OR (alfentanil or alphaprodine or buprenorphine or butorphanol or codeine or dezocine or dihydrocodeine or fentanyl or hydrocodone or hydromorphone or levomethadyl or levorphanol or meperidine or methadone or morphine or nalbuphine or opium or oxycodone or oxymorphone or pentazocine or propoxyphene or remifentanil or sufentanil or tapentadol or tramadol or heroin or nalmefene or naloxone or naltrexone)) AND ("Pregnancy"[Mesh]) AND (("Congenital Abnormalities"[Mesh]) OR (congenital malformation) OR (birth defect)) |
| Embase |
| #1 ‘pregnancy’/exp  #2 ‘congenital disorder’/exp  #3 ‘opiate’/exp  #4 opioid  #5 alfentanil OR alphaprodine OR buprenorphine OR butorphanol OR codeine OR dezocine OR dihydrocodeine OR fentanyl OR hydrocodone OR hydromorphone OR levomethadyl OR levorphanol OR meperidine OR methadone OR morphine OR nalbuphine OR opium OR oxycodone OR oxymorphone OR pentazocine OR propoxyphene OR remifentanil OR sufentanil OR tapentadol OR tramadol OR heroin OR nalmefene OR naloxone OR naltrexone  #6 congenital AND malformation  #7 birth AND defect  #8 #2 OR #6 OR #7  #9 #3 OR #4 OR #5  #10 #1 AND #8 AND #9 |
| Cochrane library of clinical trials |
| #1 Mesh descriptor: [Pregnancy] explode all trees  #2 pregnancy  #3 Mesh descriptor: [Analgesics, Opioid] explode all trees  #4 opioid  #5 alfentanil or alphaprodine or buprenorphine or butorphanol or codeine or dezocine or dihydrocodeine or fentanyl or hydrocodone or hydromorphone or levomethadyl or levorphanol or meperidine or methadone or morphine or nalbuphine or opium or oxycodone or oxymorphone or pentazocine or propoxyphene or remifentanil or sufentanil or tapentadol or tramadol or heroin or nalmefene or naloxone or naltrexone  #6 Mesh descriptor: [Congenital abnormalities] explode all trees  #7 congenital malformation  #8 birth defect  #9 (#1 OR #2) AND (#3 OR #4 OR #5) AND (#6 OR #7 OR #8) |

**Table S2** Summary of risk of bias assessment using ROBINS-I tool

|  | Bias due to confounding | Bias in selection of participants into the study | Bias in classification of interventions | Bias due to deviations from intended interventions | Bias due to missing data | Bias in measurement of outcomes | Bias in selection of the reported result | Overall bias |
| --- | --- | --- | --- | --- | --- | --- | --- | --- |
| Bateman/2021 | Moderate | Low | Low | Moderate | No information | Low | Low | Moderate |
| Wen/2021 | Moderate | Serious | Moderate | Moderate | No information | Low | Moderate | Serious |
| Fishman/2019 | Serious | Low | Moderate | Moderate | No information | Low | Low | Serious |
| Kelty/2017 | Serious | Low | Moderate | Critical | No information | Low | Low | Critical |
| Jumah/2016 | Critical | Low | Moderate | Critical | Moderate | No information | Serious | Critical |
| Kallen/2015 | Serious | Low | Serious | Moderate | No information | Low | Low | Serious |
| Norgaard/2015 | Critical | Low | Moderate | Critical | No information | Low | Low | Critical |
| Saleh Gargari/2012 | Critical | Moderate | Critical | Critical | No information | No information | Serious | Critical |
| Greig/2012 | Critical | Low | Serious | Critical | Moderate | No information | Serious | Critical |
| Nezvalová-Henriksen/2011 | Moderate | Low | Low | Moderate | Moderate | Moderate | Low | Moderate |
| Vucinovic/2008 | Critical | Serious | Critical | Critical | Moderate | No information | Serious | Critical |
| Cleary/2011 | Moderate | Serious | Moderate | Moderate | Moderate | Low | Low | Serious |
| Kallen/2013 | Moderate | Serious | Serious | Serious | No information | Low | Serious | Serious |
| Brown/1998 | Serious | Serious | Moderate | Critical | No information | No information | Serious | Critical |
| Ellwood/1987 | Critical | Serious | Critical | Critical | No information | No information | Serious | Critical |
| Wilson/1981 | Serious | Serious | Critical | Critical | Moderate | No information | Serious | Critical |
| Ostrea/1979 | Critical | No information | No information | Critical | No information | No information | Critical | Critical |
| Stimmel/1976 | Critical | Critical | Critical | Critical | No information | No information | Serious | Critical |

**Figure S1:** Eggers’s test of studies examining the association between opioids exposure and the risk of congenital malformations

**Figure S2:** Forest plot of association between opioid exposure and major congenital malformation

TE, treatment effect; SE, standard error; IV, inverse variance test; CI, confidence interval; df, degrees of freedom

**Figure S3**: Forest plot of association between opioid exposure and central nervous system malformation

TE, treatment effect; SE, standard error; IV, inverse variance test; CI, confidence interval; df, degrees of freedom

**Figure S4:** Forest plot of association between opioid exposure and limb malformation

TE, treatment effect; SE, standard error; IV, inverse variance test; CI, confidence interval; df, degrees of freedom

**Figure S5:** Forest plot of assoxiation between opioid exposure and cardiovascular malformation

TE, treatment effect; SE, standard error; IV, inverse variance test; CI, confidence interval; df, degrees of freedom

**Figure S6:** Forest plot of association between opioid exposure and gastrointestinal malformation

TE, treatment effect; SE, standard error; IV, inverse variance test; CI, confidence interval; df, degrees of freedom

**Figure S7:** Forest plot of association between opioid exposure and ear, face, and neck malformation


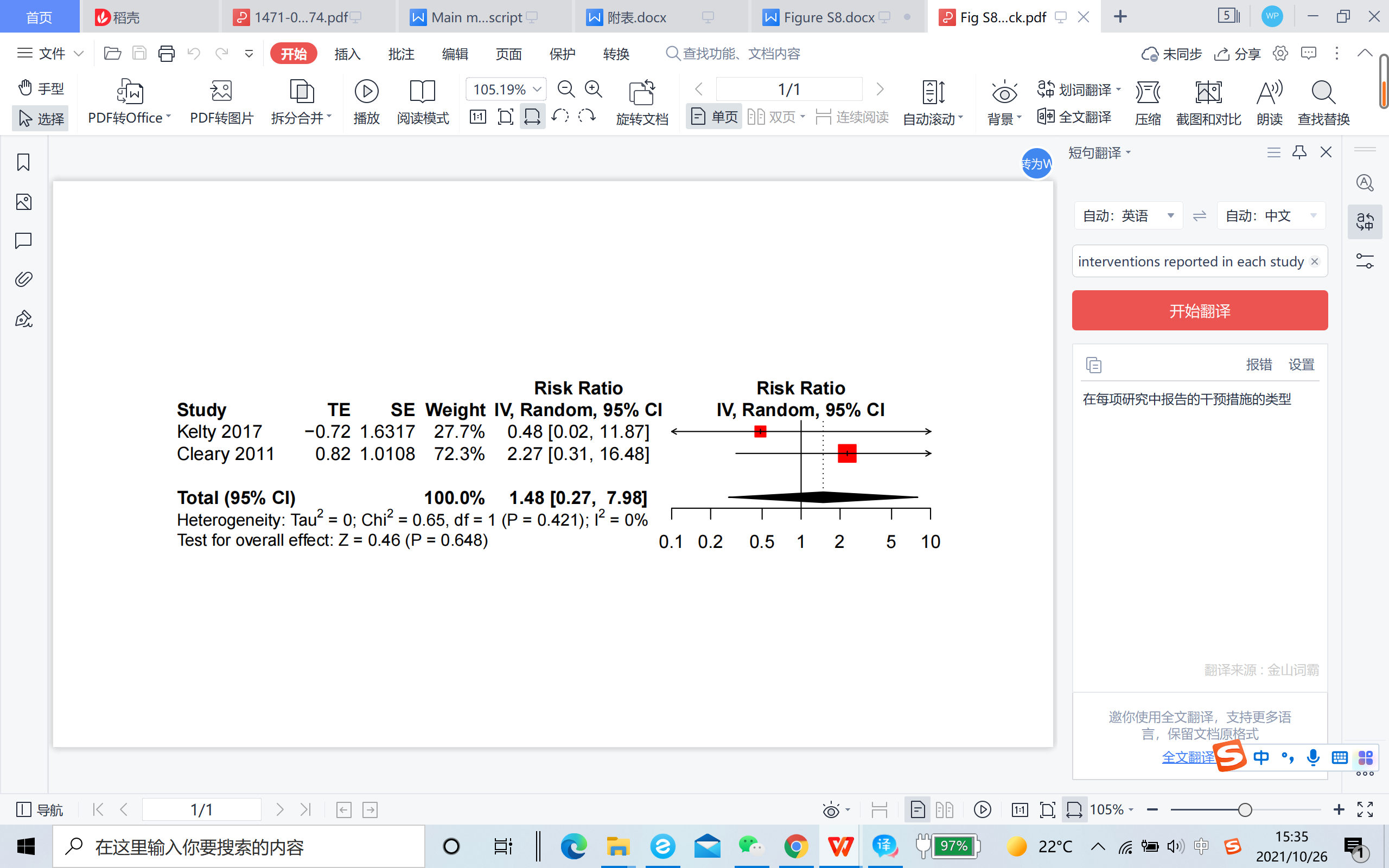


TE, treatment effect; SE, standard error; IV, inverse variance test; CI, confidence interval; df, degrees of freedom

**Figure S8:** Forest plot of association between opioid exposure and respiratory malformation


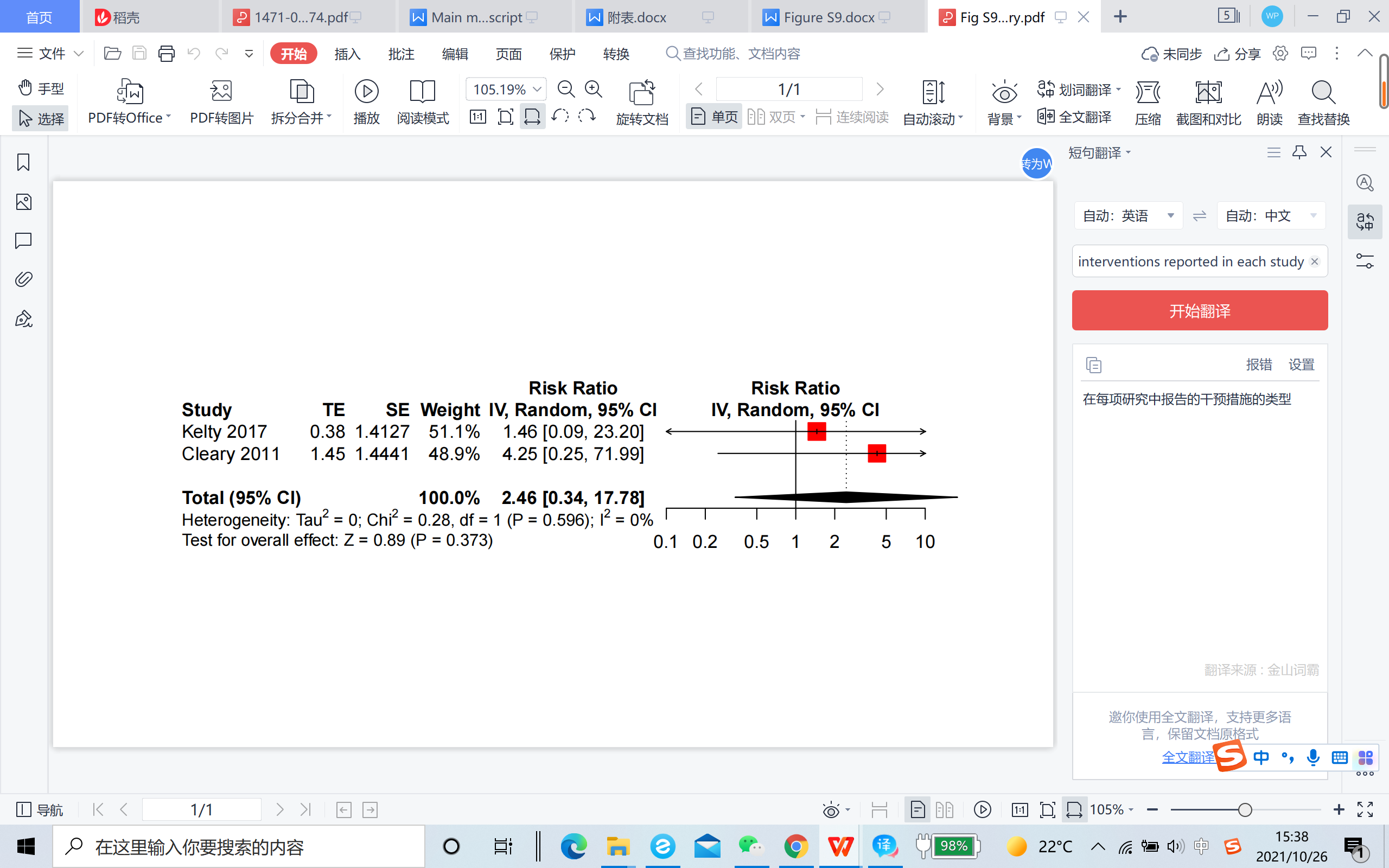


TE, treatment effect; SE, standard error; IV, inverse variance test; CI, confidence interval; df, degrees of freedom

**Figure S9:** Forest plot of association between opioid exposure and musculoskeletal malformation

TE, treatment effect; SE, standard error; IV, inverse variance test; CI, confidence interval; df, degrees of freedom

**Figure S10:** Forest plot of association between opioid exposure and urogenital malformation

TE, treatment effect; SE, standard error; IV, inverse variance test; CI, confidence interval; df, degrees of freedom

**Figure S11**: Forest plot of association between opioid exposure and orofacial malformation

TE, treatment effect; SE, standard error; IV, inverse variance test; CI, confidence interval; df, degrees of freedom

**Figure S12:** Forest plot of association between opioid exposure and neural tube defects

TE, treatment effect; SE, standard error; IV, inverse variance test; CI, confidence interval; df, degrees of freedom

**Figure S13:** Forest plot of association between opioid exposure and gastroschisis


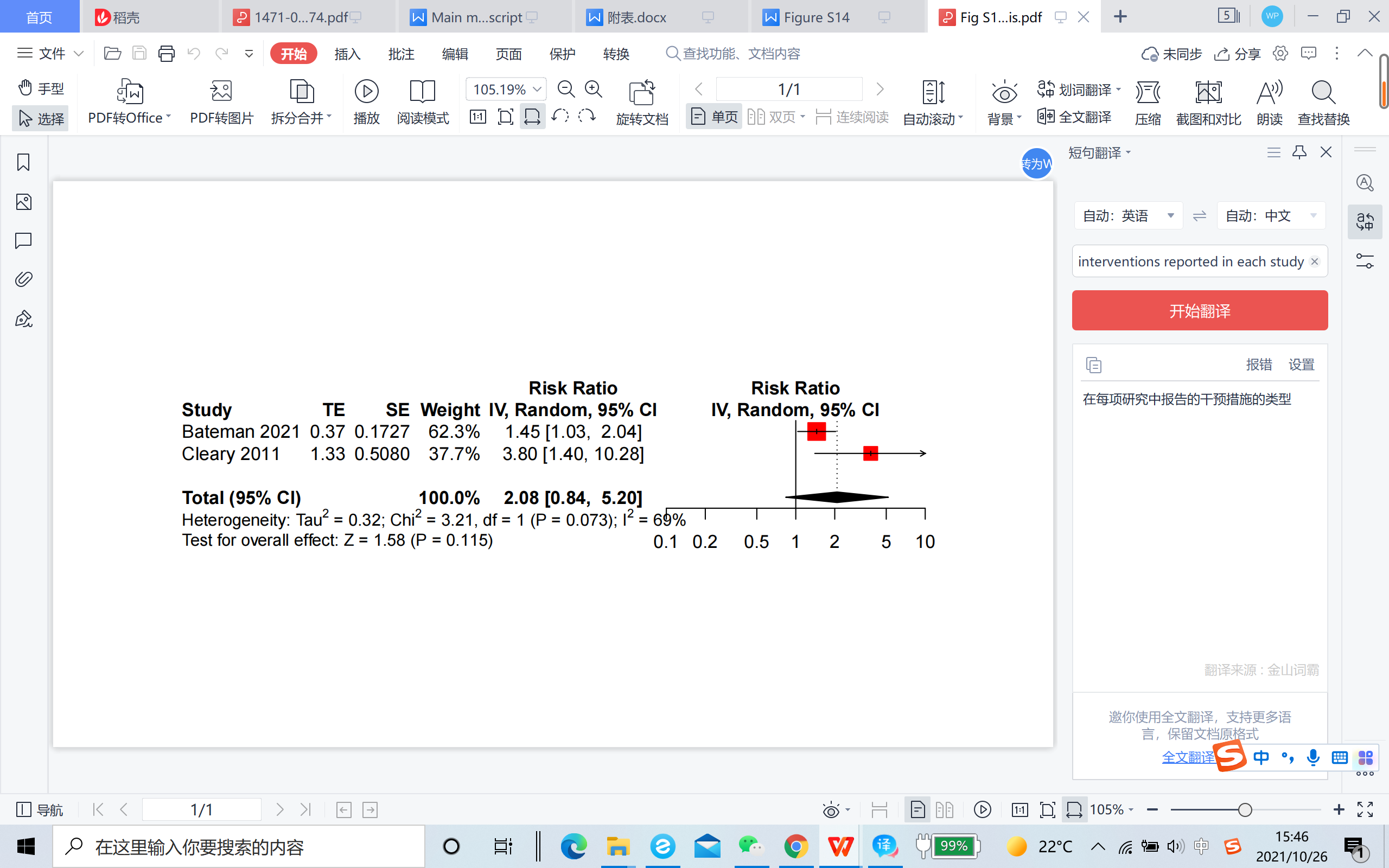


TE, treatment effect; SE, standard error; IV, inverse variance test; CI, confidence interval; df, degrees of freedom

**Figure S14:** Forest plot of association between opioid exposure and clubfoot


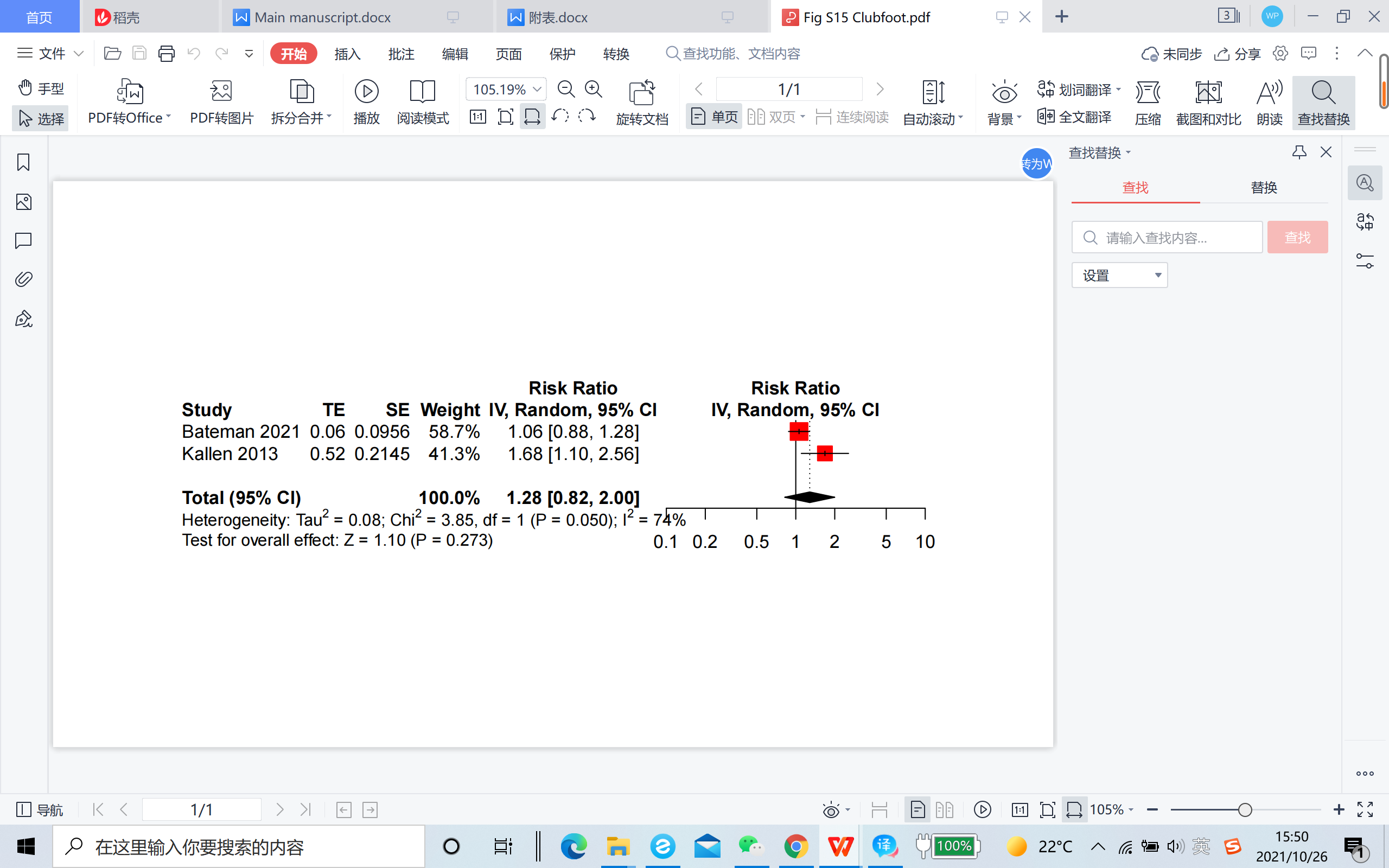


TE, treatment effect; SE, standard error; IV, inverse variance test; CI, confidence interval; df, degrees of freedom

**Figure S15:** Meta-regression according to the year of publication

**Figure S16:** Forest plot of subgroup analysis of exposed period


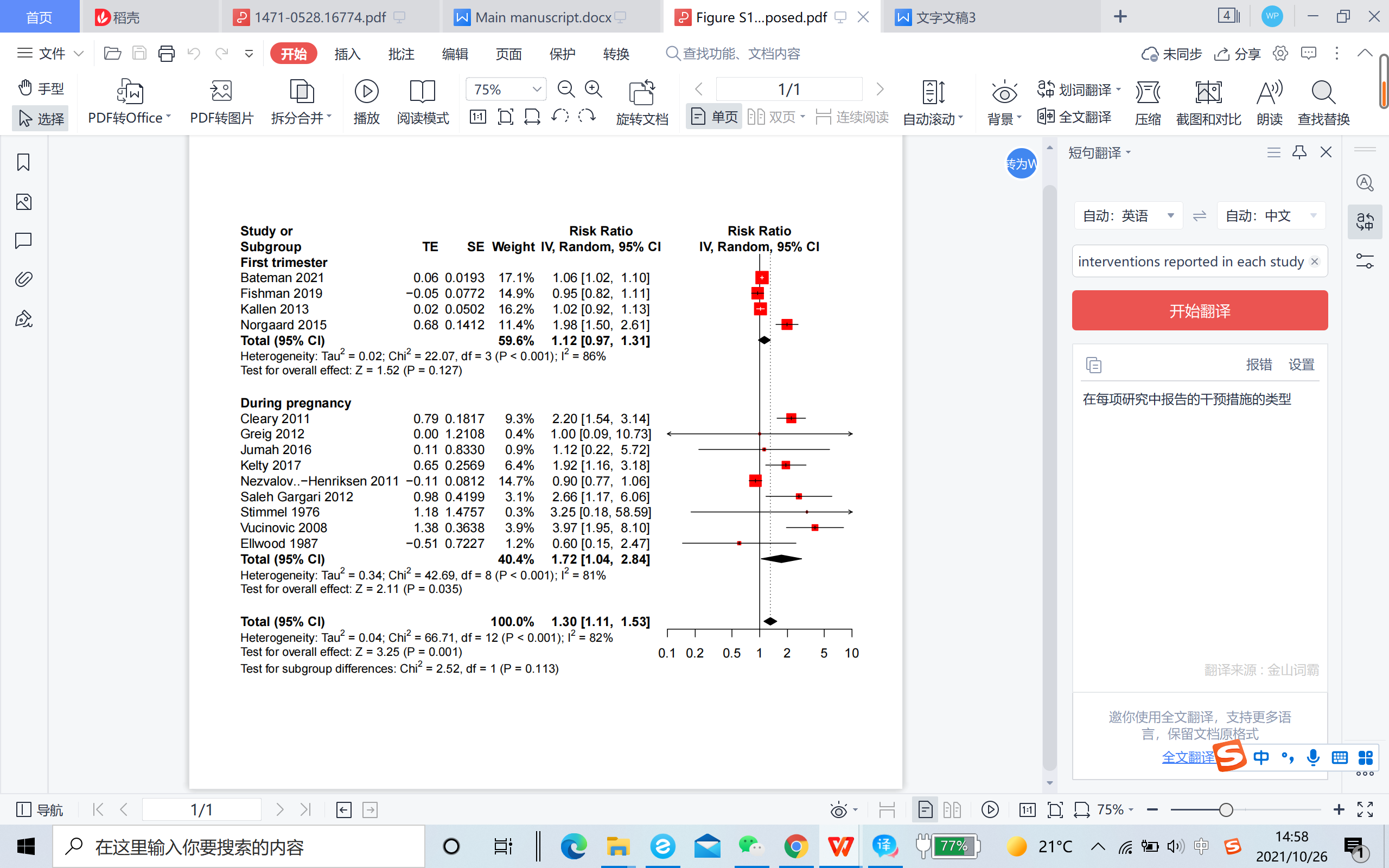


TE, treatment effect; SE, standard error; IV, inverse variance test; CI, confidence interval; df, degrees of freedom

**Figure S17:** Forest plot of subgroup analysis of indication


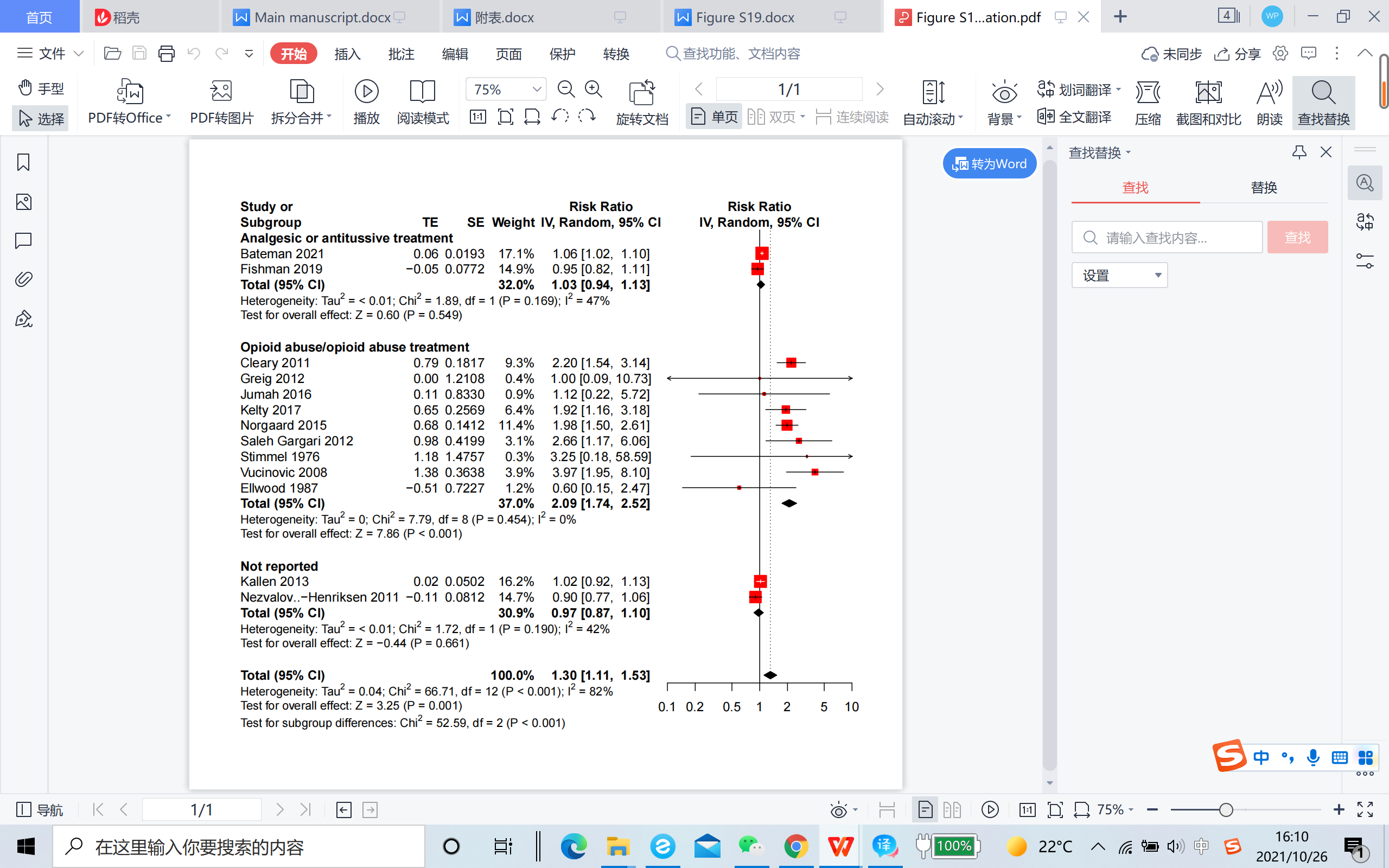


TE, treatment effect; SE, standard error; IV, inverse variance test; CI, confidence interval; df, degrees of freedom

**Figure S18:** Forest plot of subgroup analysis of adjusted for confounders


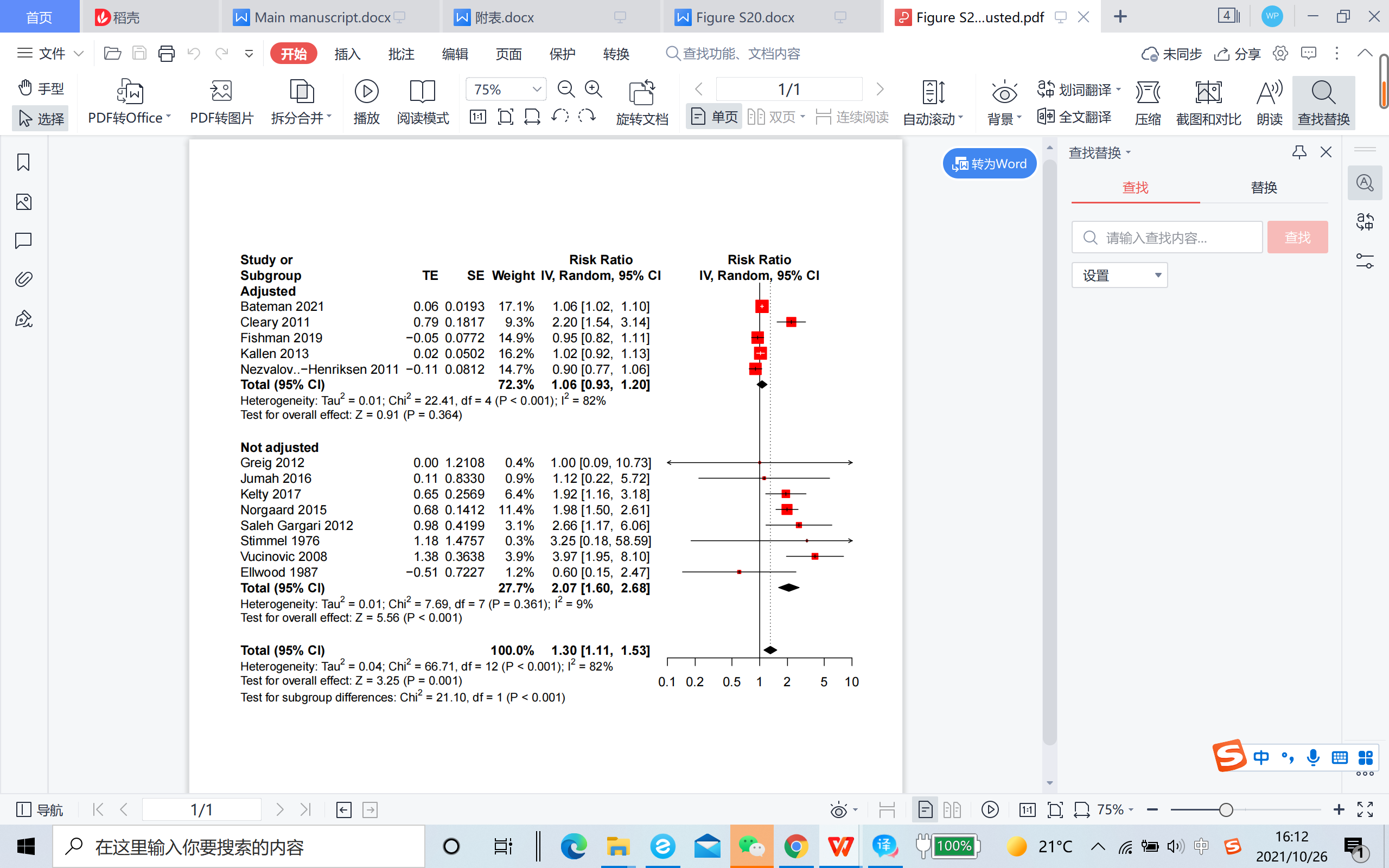


TE, treatment effect; SE, standard error; IV, inverse variance test; CI, confidence interval; df, degrees of freedom

**Figure S19:** Forest plot of subgroup analysis of risk of bias assessment

TE, treatment effect; SE, standard error; IV, inverse variance test; CI, confidence interval; df, degrees of freedom
